# Postpartum Hemorrhage in Pregnancy beyond 40 Weeks of Gestation in a Tertiary Care Hospital: A Descriptive Cross-sectional Study

**DOI:** 10.31729/jnma.6471

**Published:** 2021-05-31

**Authors:** Rashmi Bastakoti Gaire, Suman Raj Tamrakar, Anjana Singh Dongol

**Affiliations:** 1 Department of Obstetrics and Gynaecology, Dhulikhel Hospital, Dhulikhel, Nepal

**Keywords:** *complication*, *postpartum hemorrhage*, *pregnancy*

## Abstract

**Introduction:**

Postpartum hemorrhage is defined as a blood loss of 500ml or more within 24 hours after birth. It is the leading cause of maternal mortality in low-income countries and the primary cause of nearly one-quarter of all maternal deaths globally. It occurs in up to 18% of total births. Post-dated pregnancy is a high-risk pregnancy with increased maternal morbidity. This study aims to determine the prevalence of postpartum hemorrhage in pregnancy beyond 40 weeks of gestation in a tertiary care hospital.

**Methods:**

A descriptive cross-sectional study was conducted among pregnant women beyond 40 weeks in Dhulikhel hospital from October 2016 to March 2017. The study was conducted after ethical clearance from the hospital research committee (reference number#128/16). The sample size was calculated and convenient sampling was done. Statistical Package for the Social Sciences is used for analysis. Point estimate at 95% confidence interval was calculated along with frequency and percentage for binary data.

**Results:**

Out of 465 ladies enrolled in this study postpartum hemorrhage was seen in 6 (1.29%) (95% Confidence Interval = 0.267-2.31), and the mean age was 24.25+4.8. About 346 (74.4%) had a normal delivery, 104 (22.36%) had cesarean section and 15 (3.22%) had instrumental delivery.

**Conclusions:**

Postpartum haemorrhage prevalence is low among the pregnant women beyond 40 weeks compared to the standard study. Postpartum hemorrhage is the common leading cause of maternal mortality. So high-risk cases should be identified and active management should be done to reduce morbidity and mortality.

## INTRODUCTION

Postpartum haemorrhage (PPH) is defined as a blood loss of 500ml or more within 24 hours after birth.^1^ PPH is the leading cause of maternal mortality in low-income countries and the primary cause of nearly one-quarter of all maternal deaths globally.^2^ PPH occurs in up to 18% of total births.^3^ Blood loss of more than a liter is considered physiologically significant and can result in hemodynamic instability.^4^ World Health Organization defines Post-Term Pregnancy (PTP) as the pregnancy extended to or beyond 42 weeks (294 days) of gestation.^5^ About 5-10% pregnancy continues to at least 42 weeks.^6^

The incidence of PTP varies depending on the calculation based on Last menstrual period (LMP) and clinical examination, or early pregnancy ultrasound.^7,8^ Latter, has reduced the incidence of PTP by 50.0%.^7^ The prevalence of PTP in Nepal is 4.6%.^9^

The aim of this study is to find the prevalence of postpartum hemorrhage in pregnancy beyond 40 weeks of gestation in a tertiary care hospital.

## METHODS

It is a descriptive cross-sectional study conducted from October 2016 to March 2017 in the Department of Obstetrics and Gynecology among pregnant women beyond 40 weeks. The study was conducted after ethical clearance from the hospital research committee (Reference no: IRC-KUSMS#128/16). All pregnant women of 40 weeks and above gestation who came to the Obstetrics and Gynecology department of Dhulikhel hospital, meeting the inclusion criteria and giving consent were enrolled in the study. The pregnant women beyond the 40 weeks not giving consent and who didn't meet inclusion criteria were excluded from the study. The sample size was calculated by using the formula,


$$\begin{array}{ll} \text{n} & \text{= }{\text{Z}}^{\text{2}}\times \text{p}\times q\;/{\text{e}}^{\text{2}}\\ & \text{= }{\left(1.96\right)}^{\text{2}}\times 0.5\times (1-0.5)/{(\text{0.05)}}^{\text{2}}\\ & \text{= }0.9604/0.0025\\ & =384.16\\ & =384 \end{array}$$

Where,

n = minimum required sample size
Z = 1.96 at 95% Confidence Interval
p = prevalence taken 50% for maximum sample size,
q = 1-p
e = margin of error, 5%

Adding a margin of error of 10% the final sample size was 422. There were 1352 ladies admitted for delivery during the study period. Out of this, 465 ladies who had fulfilled the inclusion criteria were enrolled in this study.

Convenient sampling was done. The inclusion criteria were singleton pregnancy, vertex presentation, gestational age beyond 40 weeks (i.e. date calculated by Last menstrual period, and /or Ultrasound). Variables like age, parity, period of gestation, mode of delivery, were recorded and observed. All women were followed up till discharge. All data were analyzed by Statistical Package for the Social Sciences (SPSS) version 21 using appropriate statistical tools. Numerical data were analyzed using frequency, mean and standard deviation. Point estimate at 95% confidence interval was calculated along with frquency and percentage for binary data.

## RESULTS

Out of the total 465 women with a pregnancy beyond 40 weeks, the prevalence of PPH among these ladies was found to be 6 (1.29%) (95% Confidence Interval = 0.267-2.31).

The most common age group in which PPH is more is in between 30-34 years which includes 2 (33.33%) pregnant women beyond 40 weeks having PPH complication. The age distribution of the pregnant women beyond 40 weeks with Postpartum hemorrhage is shown in (Table 1).

**Table 1:** Age distribution with PPH complications (n = 6).

Age	n (%)
<15	0 (0)
15-19	0 (0)
20-24	1 (16.66)
25-29	1 (16.66)
30-34	2 (33.33)
35-39	1 (16.66)
≥40	1 (16.66)
Total	6 (100)

The modes of deliveries were found to be as follows (Table 2).

**Table 2:** Mode of delivery among the patients beyond 40 weeks of gestation (n = 465).

Variable	≥40 weeks n (%)
Mode of delivery	
Normal delivery	346 (74.40)
Instrumental delivery	15 (3.22)
Caesarean section	104 (22.36)

## DISCUSSION

This study was conducted at Dhulikhel Hospital, Department of Obstetrics and Gynaecology. Among 465 pregnant beyond 40 weeks, Postpartum haemorrhage (PPH) was present in 6 (1.29%) (at 95% Confidence Interval = 0.267-2.31). The finding of our study is low as compared to the study by Caughey, at al.^10^ The most important cause of PPH was due to atonicity which was also seen in other studies. Atonic PPH remains the most important cause as is seen in other studies.^11^

Though we know that PPH is more common in grand multipara by definition more than 5 deliveries, we found the prevalence to be higher in multiparas (>2 deliveries) which was similar to the above study.^12^

In the above study, there was no maternal mortality, but overall maternal morbidity was 4.9%, which is lower than reported by Pradhan, et al. 7.2%. The main complication was postpartum hemorrhage ie in 6 cases (21.42%) which is less than shown by in Pradhan, et al.^13^

In this study, the incidence of post-dated pregnancy is 46.6% and the postterm pregnancy is 3.3% which was less than those reported by other studies, 4.6% by R Marahatta in Nepal, 8.3% by Ingemarsson and Kallen in Sweden, and 7.6% by Ahanya, et al.^14-16^ The postterm pregnancy was less in the above study since in our hospital we induce the patient at 40 weeks 5 or 6 days of gestation.

In the above study, 346 had a normal delivery, 104 had Lower segment Cesarian section (LSCS), and 15 had instrumental delivery in the study group at 40-41.6 weeks of gestation. The rate of cesarean section was 22.3% in the postdated group and 19.4% at postterm pregnancy. This study was not similar to the study done by R Marahatta in his study Cesarian section (CS) was higher 15.7% among post-term pregnancy in comparison to term group 9.3%.^14^

So, to minimize the maternal and neonatal outcome and more normal delivery, we should terminate the pregnancy at 40-41.6 weeks of gestation. This study was done in a community based tertiary hospital where most of the ladies come for delivery after prolong pregnancy when they are referred from nearby health centres or come late if labour pain does not occur after the expected date. Post-dated pregnancy is a highrisk pregnancy associated with maternal morbidity. Prolonged pregnancy should be timely terminated in order to minimize complications. Since this is a descriptive cross-sectional study conducted in the single institution in the small population, the findings of the study cannot be generalized to whole population. Similar, study should be conducted in the larger setting so, that the complication in the pregnent women can be effectively managed early so that maternal mortality can be reduced in our settings.

## CONCLUSIONS

Postpartum haemorrhage prevalence is low among the pregnant women beyond 40 weeks in this study as compared to the standard study. Postpartum haemorrhage is a common complication in pregnant women. PPH is unpredictable mostly occurring without warning, one need to carry out appropriate measures in the patient who have a risk factor for PPH. So, active management of the third stage of labor should be done to prevent most of PPH. Timely identification can prevent the maternal morbidity and motility.
